# Ovarian Basaloid Carcinoma with Shadow Cell Differentiation

**DOI:** 10.1155/2014/391947

**Published:** 2014-02-03

**Authors:** Michal Zamecnik, Daniel Jando, Peter Kascak

**Affiliations:** ^1^Medicyt, s. r. o., Department of Surgical Pathology, Laboratory Trencin, Legionarska 28, 91171 Trencin, Slovakia; ^2^Agel, a. s., Laboratory of Surgical Pathology, 74101 Novy Jicin, Czech Republic; ^3^Department of Radiology, Faculty Hospital, 91171 Trencin, Slovakia; ^4^Department of Obstetrics and Gynecology, Faculty Hospital, 91171 Trencin, Slovakia; ^5^Faculty of Health, Alexander Dubcek University, 91171 Trencin, Slovakia

## Abstract

So-called shadow cell differentiation (SCD) is typical for pilomatrixoma and other skin lesions with follicular differentiation, but it was rarely described also in some visceral carcinomas. We report a case of ovarian basaloid carcinoma with SCD. The tumor presented as a 14 cm ovarian mass in a 45-year-old woman, and therefore the adnexectomy and hysterectomy were performed. The tumor was of high stage. Multiple metastases were found in the liver, retroperitoneal and mediastinal lymph nodes, and the lung. Histologically, the tumor showed a pattern of high-grade basaloid carcinoma with numerous shadow cells. Extensive histologic examination did not reveal any glandular or preexisting teratoma component. Immunohistochemically, the tumor expressed markers of squamous cell differentiation, such as p63, cytokeratin 5/6, and high-molecular-weight keratin. Cytokeratin 7 and CA125 were positive in scattered cells of the lesion. Estrogen and progesterone receptor, vimentin, and p53 were negative. Beta-catenin showed nuclear and cytoplasmic positivity, indicating possible tumor proliferation/differentiation via Wnt signaling pathway. To our knowledge, SCD in basaloid carcinoma of the ovary was not described before. In addition to the description of the case, we review the literature on SCD in visceral carcinomas.

## 1. Introduction

So-called shadow cells (ghost cells) are specialized form of cornified cells in which, as a consequence of karyolysis, nuclei have faded, but empty spaces in the sites of the nuclei still can be recognized. The cytoplasm of the shadow cells is fine, filamentous or granular, eosinophilic, often with yellowish (amber- or honey-like) shade. They are typical for pilomatrixoma and other cutaneous tumors with follicular differentiation, and it was suggested that they represent faulty attempts at differentiation toward hair [1, 2]. Shadow cell differentiation (SCD) was, however, found also in noncutaneous lesions, such as craniopharyngioma [3], odontogenic cyst [3], gonadal teratomas [4–6], and in some visceral carcinomas, including uterine and ovarian endometrioid carcinomas [7–14]. To our knowledge, SCD was not reported in high-grade basaloid carcinoma of the ovary [15], before. We would like to demonstrate an example of such tumor here.

## 2. Case Report

A 45-year-old para 2, gravida 3 patient, nonsmoker, was admitted for 3-week lasting pelvic pain. Her medical history included right adnexectomy for benign mucinous ovarian cystadenoma performed three years ago. Ultrasound scan found left-sided adnexal tumor measuring 13 × 9 cm, without apparent metastases in the pelvic lymph nodes. Adnexectomy and hysterectomy were performed, with peritoneal washing for cytological examination. After the resection, histological diagnosis of basaloid carcinoma with SCD was done. Because primary ovarian carcinoma of basaloid morphology is rare, a metastatic nature of the tumor was considered, and therefore it was recommended to perform work-up directed toward exclusion of nongynecologic primary carcinoma (especially of carcinoma in the lung, head and neck region, or in the skin). CT scans showed multiple metastases in the retroperitoneal and mediastinal lymph nodes, liver, and lung. An extensive search for primary tumor in any nonovarian locations gave negative results, and the diagnosis of ovarian basaloid carcinoma with SCD was rendered finally. Chemotherapy was planned but not started because patient's state worsened gradually due to tumor generalization with associated hepatic and renal failure. She died 8 weeks after the surgery. Autopsy was not performed.

Grossly, a 14 cm measuring adnexal tumor showed glossy external surface (tumor capsule). The cut surface was solid and vaguely lobular. Its color was grey, with numerous yellowish necroses. Some of the necroses were pseudocystic.

**Figure 1:**
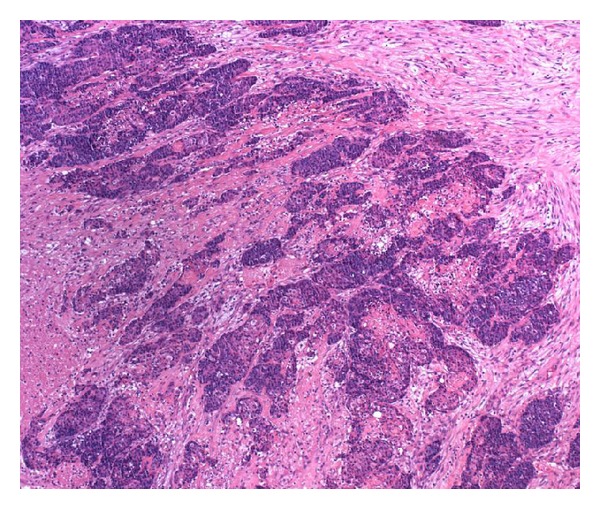
At low-power, dark basaloid cell clusters in desmoplastic stroma and necroses are seen (hematoxylin and eosin).

**Figure 2:**
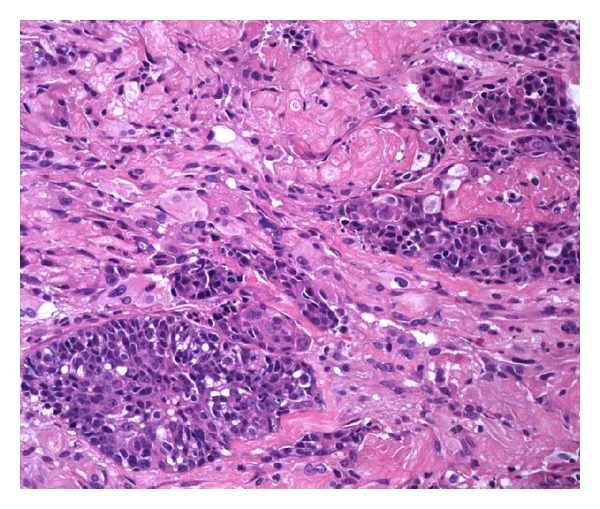
Basaloid morphology of the tumor, with numerous keratinized shadow cells (hematoxylin and eosin).

**Figure 3:**
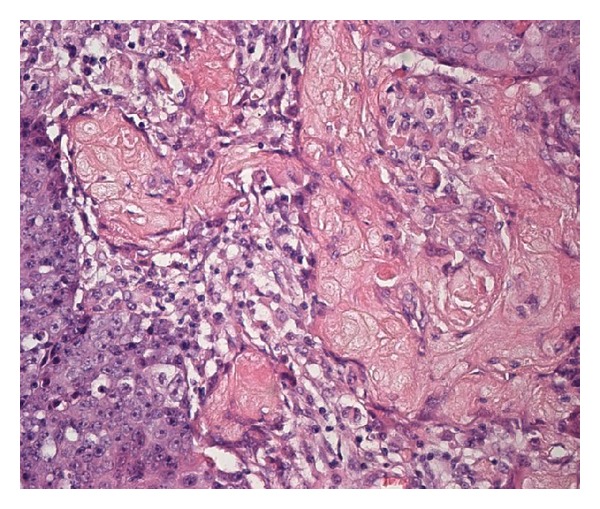
The cytoplasm of the shadow cells is fine and granular, eosinophilic, with yellowish (honey-like) shade. Some shadow cell clusters were isolated in the inflamed stoma (hematoxylin and eosin).

**Figure 4:**
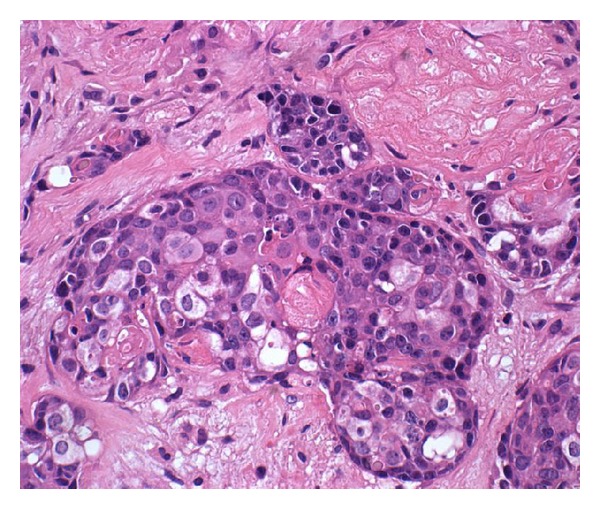
In addition to basaloid and shadow cells, transitional cells with eosinophilic to clear cytoplasm were seen in some cell clusters (hematoxylin and eosin).

Histologically, the tumor showed features of high-grade basaloid carcinoma with keratinization [15, 16]. It was composed of basaloid cells growing in solid clusters in desmoplastic stroma (Figure 1). The tumor cell islands showed frequent necrosis, which was focally confluent and extensive. Nuclear atypia and pleomorphism were intense, and mitotic figures were frequent. Many cell clusters contained keratinized shadow cells or debris of keratin (Figures 2 and 3). The transition between shadow cells and basaloid appearing cells was either abrupt or gradual. In foci of gradual transition, squamous cells with eosinophilic to clear cytoplasm were seen between shadow cells and basaloid cells (Figure 4). In some areas, isolated shadow cell nests were observed in the desmoplastic stroma, sometimes with foreign-body giant-cell reaction. Rare histological sections contained remnants of tubal wall infiltrated by the tumor. Stains for mucin were negative in the tumor cells, and extensive sampling (80 tissue blocks) did not reveal any glandular element or any tissue of the teratoma. In the uterus, the findings included hypoproliferative endometrium and cervical squamous metaplasia, without any significant atypia.

**Figure 5:**
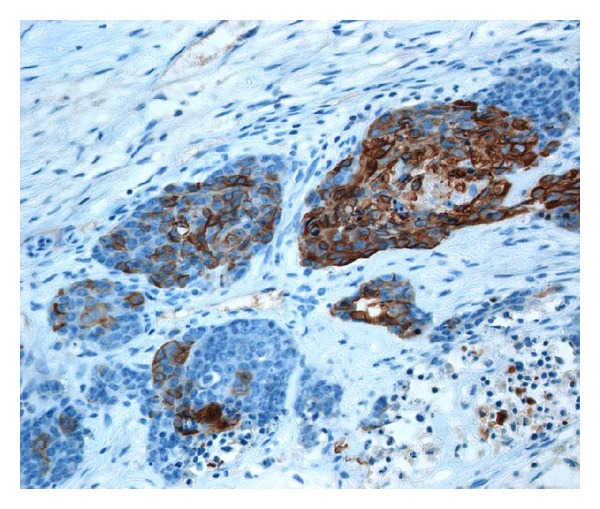
Immunohistochemical expression of high-molecular-weight cytokeratin (SABC technique).

**Figure 6:**
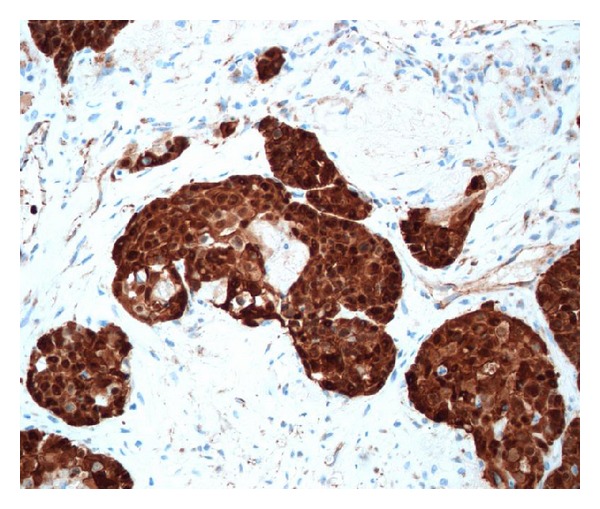
Positivity for beta-catenin is seen in both cytoplasm and nuclei of the neoplastic cells, except of dead shadow cells (SABC technique).

**Figure 7:**
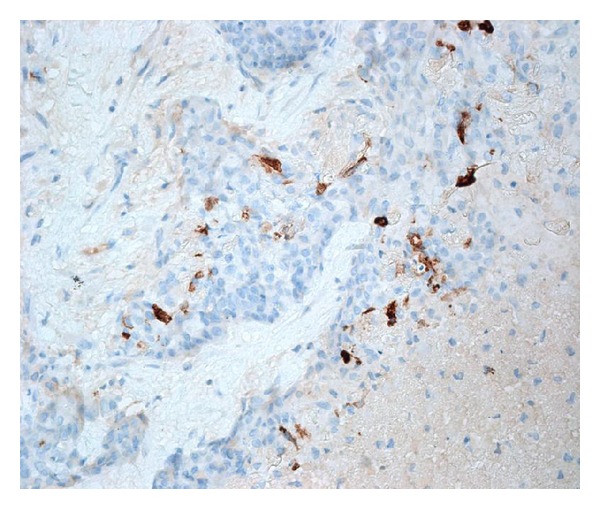
CA125 is positive in scattered tumor cells (SABC technique).

Immunohistochemically, the tumor was positive for pancytokeratin, high-molecular-weight keratin (Figure 5), CEA, cytokeratin 5/6, and p63. Beta-catenin (Figure 6) showed cytoplasmic staining in all cells except of shadow cells. Nuclear staining was limited mostly to the basaloid cells, whereas the nuclei of squamous eosinophilic to clear cells were usually negative or mildly positive. Shadow cells were beta-catenin-negative. CA125 was positive in scattered cells of the tumor (Figure 7). The following antibodies gave negative results in the tumor cells: vimentin, p16, p53, cytokeratin 7 (CK7), cytokeratin 20, estrogen receptor (ER), progesterone receptor (PR), TTF-1, uroplakin III, chromogranin A, CD56, and synaptophysin.

Peritoneal fluid cytology found mesothelial cells and lymphocytes, and it was negative for tumor cells.

## 3. Discussion

The present basaloid carcinoma was composed of pattern typical for this entity, that is, nests of small to medium sized atypical cells with hyperchromatic nuclei and scant cytoplasm, often with peripheral palisading [15, 16]. In addition, it contained numerous shadow cells which were quite similar to those described in visceral carcinomas with SCD [7–14]. These cells are fully cornified, with fine filamentous or granular eosinophilic cytoplasm. Empty spaces in the sites of the nuclei are irregular in both size and shape, reflecting the nuclear pleomorphism of the preexisting atypical nuclei (and contrasting with quite regular empty spaces seen in benign tumors with SCD, e.g., in cutaneous pilomatrixoma).

In the ovarian location, the shadow cells were described in benign pilomatrixoma in dermoid cyst (mature teratoma) [5] and in endometrioid carcinoma [9, 12], but they were not reported in basaloid carcinoma [15]. For this reason, we performed extensive sampling and we searched for preexisting teratoma or for pattern of endometrioid carcinoma (with negative result) before our final diagnosis of “pure” basaloid carcinoma was rendered. Basaloid carcinoma of the ovary is rare [15]. Majority of cases contained, in addition to basaloid or basaloid-squamous pattern, also a minor glandular component. Therefore, Eichhorn and Scully suggest that these tumors represent an unusual pattern of growth in surface epithelial-stromal tumors rather than an independent tumor type [15]. However, some basaloid carcinomas including our case lack glandular component, and thus their histogeneses remain unclear. Although glandular differentiation was not apparent in our case by conventional histological examination, we speculated that it could be minimal and visible only at the immunohistochemical level. Therefore, we performed immunostains which could support mullerian-type differentiation, such as ER, PR, CK7, and CA125 [17]. ER and PR were negative, and CK7 and CA125 were positive in only a few cells. Such finding is insufficient for evidence of mullerian or surface epithelial differentiation, and thus histogenesis of the lesion in our case remains uncertain.

As mentioned above, SCD is not limited to cutaneous, odontogenic, and teratomatous tumors, but it was described also in rare visceral carcinomas (together 13 reported cases; for overview, see Table 1). The sites of these carcinomas include ovary [9], uterus [7], colon [7, 14], lung [13], gallbladder [10], and urinary bladder [8, 11]. In our experience, SCD is quite often seen in endometrioid carcinomas with squamous cell metaplasia. These cases are substantially more frequent than it appears due to the rarity of published cases, and SCD in endometrioid carcinoma is usually considered by pathologists to be a squamous cell metaplasia with keratinization. We think that, for example, Kim and Scully's paper on peritoneal keratin granulomas in cases of ovarian and endometrial carcinomas pictures shadow cells (Figure 4 of this paper) [18].

The shadow cells represent “dead” cells, and thus SCD can be regarded as a mode of terminal differentiation. Nakamura [19] studied apoptosis-related markers in pilomatrixoma. He found that SCD is not identical to apoptosis, and that it represents probably “apoptosis-like programmed cell death” after classification by Nakamura [19], Leist and Jäättelä [20]. In our case, we observed strong expression of beta-catenin in nuclei as well as in cytoplasm of the tumor cells. Whereas this expression is known in pilomatrixomas [21], only one case of visceral carcinoma with SCD was examined for beta-catenin previously [8]. It was urothelial carcinoma with SCD, and this tumor was strongly beta-catenin-positive in contrast with urothelial carcinomas with “conventional” squamous cell differentiation (lacking shadow cells) [8]. In tumorigenesis, beta-catenin in the nucleus acts as a key component in so-called Wnt signal transduction pathway [22]. Its nuclear positivity in tumors with SCD indicates that SCD could represent morphologic reflection of this molecular abnormality.

In conclusion, we described a rare case of ovarian basaloid carcinoma with SCD. The shadow cells were similar to those described in visceral adenocarcinomas with SCD previously. Although majority of ovarian basaloid carcinoma are of ovarian surface epithelium origin [15], the histogenesis in the present case remains unclear because the tumor did not contain any glandular component and it did not express immunohistochemical markers of ovarian surface/mullerian differentiation. In addition, no remnant of teratoma was found, and thus teratomatous origin of the tumor appears to be improbable. Beta-catenin nuclear expression observed in the present tumor and in one previously reported case [8] indicates that beta-catenin can play significant role in proliferation and differentiation of carcinomas with SCD via the Wnt signaling pathway.

## Figures and Tables

**Table 1:** Reported cases of visceral carcinoma with shadow cell differentiation.

Primary site	Age/sex	Histological type	Reference	Year
Ovary	48/F	Adenosquamous carcinoma	[9]	1996
Ovary	31/F	Endometrioid carcinoma (skin metastasis)	[12]	2010
Ovary	45/F	High-grade basaloid carcinoma	Present case	2013
Uterus	40/F	Endometrioid with squamous differentiation	[7]	1995
Uterus	53/F	Endometrioid with squamous differentiation	[7]	1995
Uterus	46/F	Endometrioid with squamous differentiation	[7]	1995
Colon	63/M	Adenocarcinoma with squamous differentiation	[7]	1995
Colon	58/M	Adenosquamous carcinoma	[7]	1995
Colon	65/M	Adenocarcinoma with squamous metaplasia	[14]	1997
Gallbladder	67/F	Small cell carcinoma	[10]	1998
Bladder	75/M	Urothelial carcinoma with squamous metaplasia	[11]	1996
Bladder	72/M	Urothelial carcinoma with squamous differentiation	[8]	2012
Lung	76/M	Squamous cell carcinoma	[13]	2002

F: female, M: male.
